# Bioinformatic analysis reveals hub genes and pathways that promote melanoma metastasis

**DOI:** 10.1186/s12885-020-07372-5

**Published:** 2020-09-07

**Authors:** Wenxing Su, Yi Guan, Biao Huang, Juanjuan Wang, Yuqian Wei, Ying Zhao, Qingqing Jiao, Jiang Ji, Daojiang Yu, Longjiang Xu

**Affiliations:** 1 Department of Dermatology, The Second Affiliated Hospital of Soochow University, No. 1055 Sanxiang Street, Suzhou, Jiangsu 215000 People’s Republic of China; 2 Department of Medicine, Soochow University, No. 199 Renai Street, Suzhou, Jiangsu 215000 People’s Republic of China; 3 School of Foreign Languages, Soochow University, No. 1 Shizi Street, Suzhou, 215000 Jiangsu People’s Republic of China; 4 Department of Burn and Plastic Surgery, The First Affiliated Hospital of Soochow University, No. 188 Shizi Street, Suzhou, Jiangsu 215000 People’s Republic of China; 5 Department of Dermatology, The First Affiliated Hospital of Soochow University, No. 188 Shizi Street, Suzhou, Jiangsu 215000 People’s Republic of China; 6 Department of Plastic Surgery, The Second Affiliated Hospital of Soochow University, No. 1055 Sanxiang Street, Suzhou, Jiangsu 215000 People’s Republic of China; 7 Department of Pathology, The Second Affiliated Hospital of Soochow University, No. 1055 Sanxiang Street, Suzhou, 215000 Jiangsu People’s Republic of China

**Keywords:** Melanoma metastasis, Bioinformatic analysis, Differentially expressed genes, Biomarker

## Abstract

**Background:**

Melanoma has the highest mortality rate of all skin tumors, and metastases are the major cause of death from it. The molecular mechanism leading to melanoma metastasis is currently unclear.

**Methods:**

With the goal of revealing the underlying mechanism, three data sets with accession numbers GSE8401, GSE46517 and GSE7956 were downloaded from the Gene Expression Omnibus (GEO) database. After identifying the differentially expressed gene (DEG) of primary melanoma and metastatic melanoma, three kinds of analyses were performed, namely functional annotation, protein-protein interaction (PPI) network and module construction, and co-expression and drug-gene interaction prediction analysis.

**Results:**

A total of 41 up-regulated genes and 79 down-regulated genes was selected for subsequent analyses. Results of pathway enrichment analysis showed that extracellular matrix organization and proteoglycans in cancer are closely related to melanoma metastasis. In addition, seven pivotal genes were identified from PPI network, including CXCL8, THBS1, COL3A1, TIMP3, KIT, DCN, and IGFBP5, which have all been verified in the TCGA database and clinical specimens, but only CXCL8, THBS1 and KIT had significant differences in expression.

**Conclusions:**

To conclude, CXCL8, THBS1 and KIT may be the hub genes in the metastasis of melanoma and thus may be regarded as therapeutic targets in the future.

## Background

Skin melanoma accounts for only 2% of skin cancers. However, due to its high malignancy and aggressiveness, it caused more than 72% of cutaneous carcinoma deaths [1]. In recent years, it has been found that some genes are closely related to the metastasis of melanoma. Previous study confirmed NEDD9 as a bona fide melanoma metastasis gene, which enhances invasion in vitro and metastasis in vivo of both normal and transformed melanocytes and interacts with focal adhesion kinase and modulated focal contact formation, showing more frequent positive overexpression in metastatic melanoma than in primary melanoma [2]. In addition, studies have shown that BRAF and NRAS mutant melanomas have similar metastasis rates and they are slightly more likely to metastasize than BRAF and NRAS wild-type melanomas [3, 4]. According to other studies, up to 85% of TERT promoter mutations have been found in metastatic melanoma, while 30–40% of TERT promoter mutations have been found in primary melanoma [5]. However, the exact molecular mechanisms that promote melanoma metastasis remain less clear.

Gene chip or gene profile is a gene detection technique that has been used for more than a decade. Using gene chips can quickly detect the expression information of all the genes within the same sample time-point, which is well suited for screening differentially expressed genes [6]. However, it is difficult to achieve reliable results due to the high false positive rate of independent chip analysis. Therefore, in this study, three mRNA microarray data sets were downloaded from the Gene Expression Omnibus (GEO) for identifying differentially expressed gene (DEG) which promotes melanoma metastasis. Then, gene ontology and pathway enrichment analysis and protein-protein interaction (PPI) network analysis were performed to help us understand the molecular mechanisms of melanoma metastasis. In conclusion, a total of 120 DEGs and three hub genes, which might play an important role in the metastasis of melanoma, were identified.

## Methods

### Data collection

GEO (http://www.ncbi.nlm.nih.gov/geo) [7] is a gene expression database created by NCBI, which contains high-throughput gene expression data submitted by research institutes worldwide. Three microarray datasets (GSE8401, GSE46517, and GSE7956) were downloaded from it (Affymetrix GPL96 platform, Affymetrix [HG-U133A] Affymetrix Human Genome U133A Array). The GSE8401 dataset includes 31 primary melanoma samples and 52 metastatic melanoma samples. GSE46517 consists of 31 primary melanoma samples and 73 metastatic melanoma samples. GSE7956 contains 10 poorly metastatic melanoma samples and 29 highly metastatic melanoma samples.

### Identification of DEGs

The DEGs between metastatic melanoma and primary melanoma samples were screened via GEO2R (http://www.ncbi.nlm.nih.gov/geo/geo2r). GEO2R is a web-based tool where users can compare two or more datasets in a GEO series in order to identify DEGs across experimental conditions [8]. The adjusted *P*-value (adj. P-value) using Benjamini and Hochberg false discovery rate were applied to discover statistically significant genes while false-positive results corrected. Probe sets with no corresponding gene symbols or genes with more than one probe set were removed or averaged, respectively. LogFC (fold change) > 0.5 and adj. P-value < 0.05 was considered statistically significant.

### Enrichment analyses of DEGs

DAVID 6.8 (https://david.ncifcrf.gov/) [9] was used for enrichment analyses to investigate DEGs at the molecular and functional level. DAVID is a comprehensive bioinformatics analysis tool, providing a set of functional annotation tools for researchers to analyze the biological functions of massive genes. In addition, FunRich [10], an open-access software enabling functional enrichment analysis and interaction network analysis of genes and proteins, was used to analyze the biological pathways of DEGs. Further evaluation of the pathway enrichment analyses of DEGs was implemented by KOBAS 3.0 (http://kobas.cbi.pku.edu.cn) [11], which annotates the input gene set with putative pathways by mapping to genes with known annotations from 5 pathway databases (KEGG PATHWAY, PID, BioCyc, Reactome and Panther). *P*-value < 0.05 was considered significant.

### PPI network construction and hub genes selection and analyses

Search Tool for the Retrieval of Interacting Genes (STRING; http://string-db.org) (version 10.0) [12], an online database of known and predicted protein interactions, was applied to predict the PPI network of DEGs. Interactions with a combined score over 0.4 were considered statistically significant. Cytoscape (http://www.cytoscape.org) (version 3.6.1) was applied to visualize the molecular interaction networks [13], by using its plug-in CytoNCA to analyze the topology characteristics of nodes in the PPI network with the parameters set as unweighted [14]. Important nodes in protein interactions within the network were obtained by ranking each node according to its score. Considering most networks were scale-free, the hub genes were selected with degrees ≥10. The enrichment analysis of biological processes was through Metascape (https://metascape.org) [15], which is an online platform specialized in comprehensive gene annotation and analysis resource. The pathway enrichment analyses for the genes were conducted by KOBAS 3.0. Besides, a network of genes and their co-expression genes was analyzed via GeneMANIA (http://www.genemania.org/) [16], which is a convenient web portal for analyzing gene lists and predicting gene function. Finally, Drug-Gene Interaction database (DGIdb) 3.0 (http://www.dgidb.org/) [17], which helps to predict drug-gene interaction networks, was adopted here to predict drugs based on the module genes, with the parameters set as following: preset filters: FDA approved; antineoplastic; all the default. After the prediction of drug-gene pairs associated with the module genes, the network map was then formed by Cytoscape.

### Validation of hub genes expression in TCGA databases

The mRNA expression of identified hub genes was verified using TCGA data which contains 102 primary melanomas and 369 metastatic melanomas (https://tcga-data.nci.nih.gov/tcga/). The comparison between the two sets of data was performed with the T-test. *P*-value < 0.05 was considered significant.

### Patients and ethical approval

From March 2016 to June 2020, a total of 72 patients from the Department of Plastic Surgery of the Second Affiliated Hospital of Soochow University obtained 36 primary cutaneous melanomas and 36 metastatic melanomas. No radiotherapy or chemotherapy was received before surgery. Intraoperative specimens of primary skin melanoma and metastatic melanoma were collected and fixed with 4% paraformaldehyde. Clinical data can be obtained from hospital records. The informed consent of all patients has been obtained before the operation, and the procedures for organizing collection have been approved by the Ethics Committee of the Second Affiliated Hospital of Soochow University, China. All procedures comply with the guidelines and ethical principles outlined in the Helsinki Declaration.

### Immunohistochemistry (IHC)

Sections were deparaffinized in xylene for 15 min and then rehydrated in graded alcohols and water. Endogenous peroxidase activity was blocked by treatment with 3% H_2_O_2_-methanol for 30 min at room temperature. After blocking nonspecific binding with serum for 40 min at 37 °C, sections were incubated with rabbit anti-CXCL8 polyclonal antibody (1:500; 27,095–1-AP), anti-THBS1 antibody (1:500; 18,304–1-AP) and anti-KIT antibody (1:500; 18,696–1-AP) in a humid chamber at 4 °C overnight. After three washes with PBS, sections were incubated with biotinylated secondary antibodies (SA00004–1, Proteintech) for 30 min. Then, they were washed three times in PBS and incubated with streptavidin-conjugated peroxidase (ab7403, Abcam) for 30 min. Slides were rinsed in PBS, exposed to diaminobenzidine (SK4100, Vector Laboratories) and counterstained with Mayer’s hematoxylin (ab128990, Abcam; negative control = omission of the primary antibody). The digital images of the specimens were scanned and obtained by the digital pathology slice scanner (DMC-10-Pro; Dmax Corporation, Suzhou, China). The percentages of cells that express CXCL8, THBS1 or KIT were assessed by quantitative histomorphometry (Olympus X71-F22PH; Olympus Corporation, Tokyo, Japan). Two experienced pathologists independently assessed the positive or negative staining of a protein in one FFPE slide and were supervised by a clinician. Based on the level of staining intensity (no staining, weak staining, medium staining and strong staining), the score ranged from 0 to 3. Based on the coverage of immunoreactive tumor cell (0%, 1–25%, 26–50%, 51–75%, 76–100%), the score was given from 0 to 4 respectively. IHC results were assigned by multiplying the score for staining intensity and the score for tumor cell area, ranging from 0 to 12 (0 to 4, negative staining; 5 to 12, positive staining).

## Results

### Identification of DEGs

**Fig. 1:**
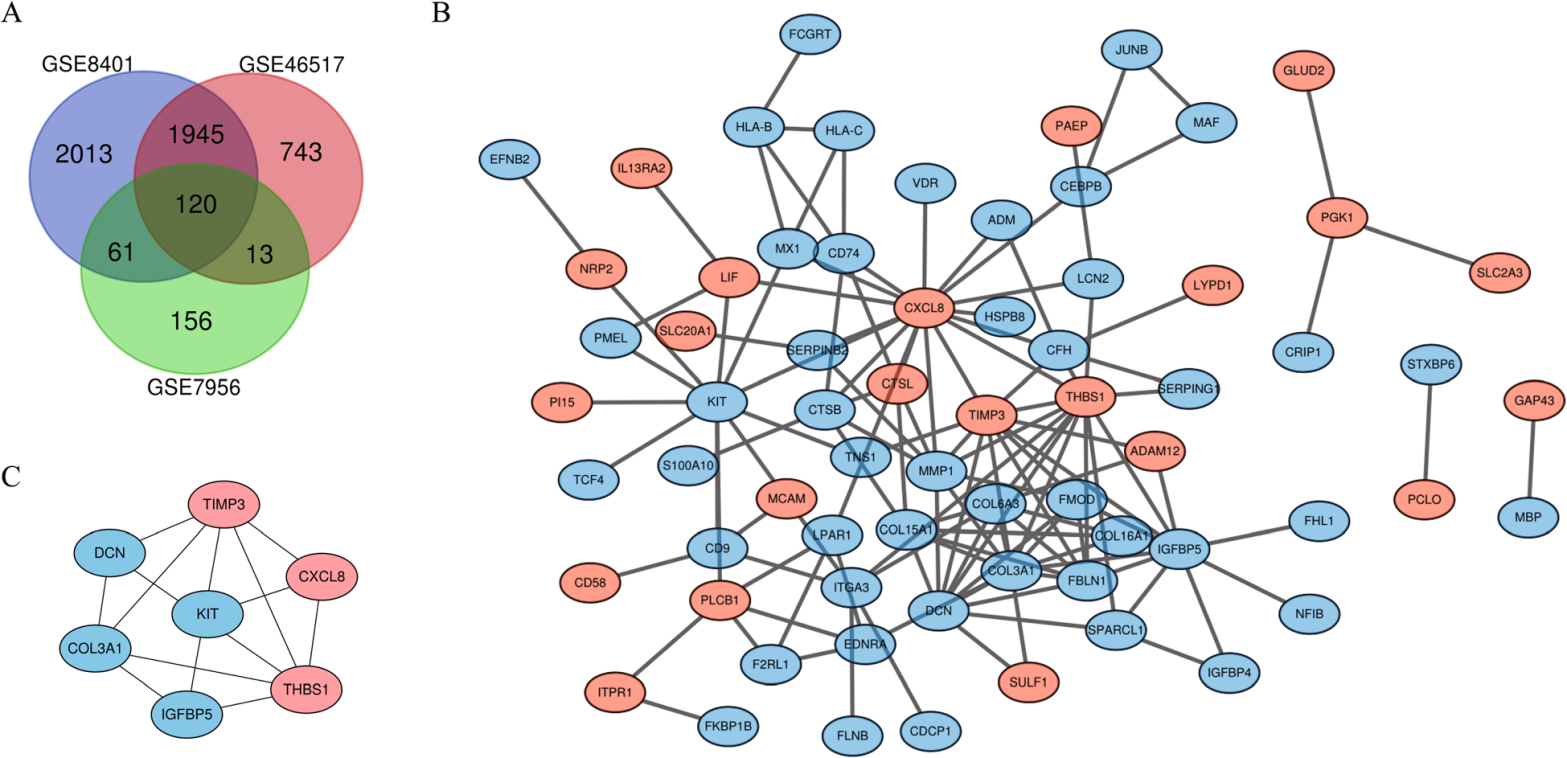
Venn diagram, PPI network and the most significant module of DEGs. **a** DEGs were selected with a fold change > 0.5 and *P*-value < 0.05 among the mRNA expression profiling sets GSE8401, GSE46517 and GSE7956. The 3 datasets showed an overlap of 120 genes. **b** The PPI network of DEGs was constructed using Cytoscape. **c** The most important module composed of seven hub genes. Upregulated genes are marked in light red; downregulated genes are marked in light blue

After standardizing the microarray results, DEGs (4139 in GSE8401, 2821 in GSE46517 and 350 in GSE7956) were identified. A total of 120 genes overlapped among the three datasets as shown in the Venn diagram (Fig. 1a), consisting of 79 downregulated genes and 41 upregulated genes.

### Analysis of the functional characteristics of DEGs

**Fig. 2:**
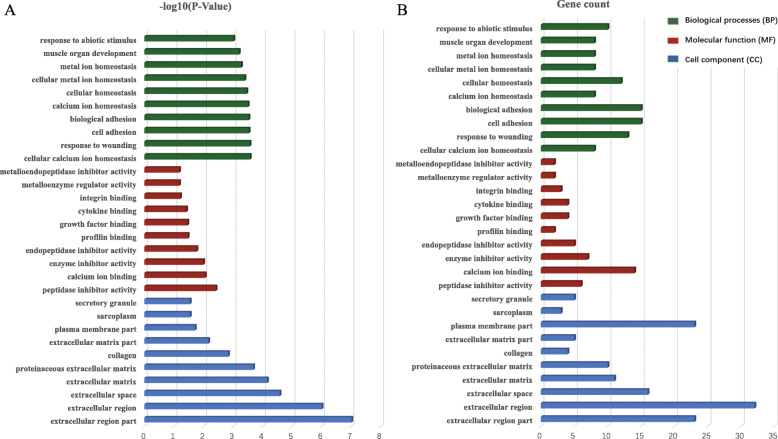
Gene Ontology analyses of differentially expressed genes (DEGs) between primary melanomas and metastatic melanomas. The biological process in functional enrichment of DEGs was performed using the online biological tool DAVID between primary melanomas and metastatic melanomas with *P* value (**a**) and gene count (**b**)

**Fig. 3:**
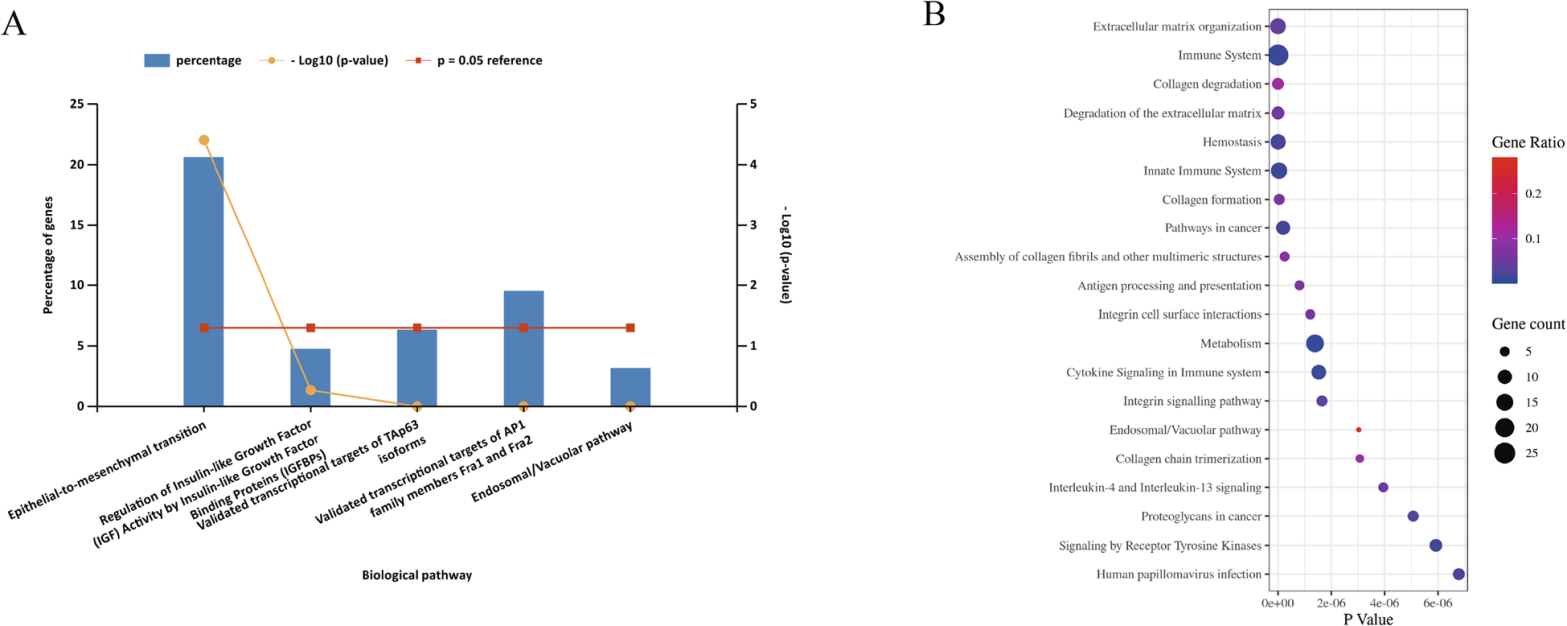
**a** The Funrich software drew a bar chart of five biological pathways based on the *P*-value and the percentage of genes, among which biological pathways with *P*-value < 0.05 are statistically significant. The results showed that the biological pathway with significantly enriched was epithelial-to-mesenchymal transition. **b** The pathway analysis of all the DEGs by KOBAS 3.0. The abscissa represents the *P*-value, and the ordinate represents the terms. The size of the circle represents the number of genes involved, and the color represents the frequency of the genes involved in the term total genes

To determine the biological functions of the above mentioned DEGs, GO enrichment analysis was performed. Results were divided into three functional categories, including biological processes (BP), cell component (CC), and molecular function (MF) (Fig. 2). For BP, DEGs were mainly enriched in cellular calcium ion homeostasis (*P* = 2.63 × 10^− 4^), response to wounding (*P* = 2.67 × 10^− 4^), cell adhesion (*P* = 2.88 × 10^− 4^) and biological adhesion (*P* = 2.92 × 10^− 4^). In terms of CC, the genes were mainly enriched in extracellular region part (*P* = 9.52 × 10^− 8^), extracellular region (*P* = 9.66 × 10^− 7^), extracellular space (*P* = 2.58 × 10^− 5^) and extracellular matrix (*P* = 6.86 × 10^− 5^). In the MF group, DEGs were significantly enriched in peptidase inhibitor activity (*P* = 3.8 × 10^− 3^) and calcium ion binding (*P* = 8.73 × 10^− 3^). Funrich analysis of enriched biological pathway for DEGs metastasizing in melanoma showed that the DEGs were mainly enriched in the epithelial-to-mesenchymal transition (EMT), as shown in Fig. 3a. According to the pathway analysis results from online database KOBAS 3.0, pathways with the top five *P*-values were extracellular matrix organization (*P* = 1.11 × 10^− 13^), immune system (*P* = 1.22 × 10^− 12^), collagen degradation (*P* = 1.54 × 10^− 10^), degradation of the extracellular matrix (*P* = 9.22 × 10^− 10^) and hemostasis (*P* = 7.92 × 10^− 9^) (Fig. 3b). These results indicate that EMT and extracellular matrix organization play an important role in the metastasis of melanoma.

### PPI network construction and hub genes selection and analysis

**Fig. 4:**
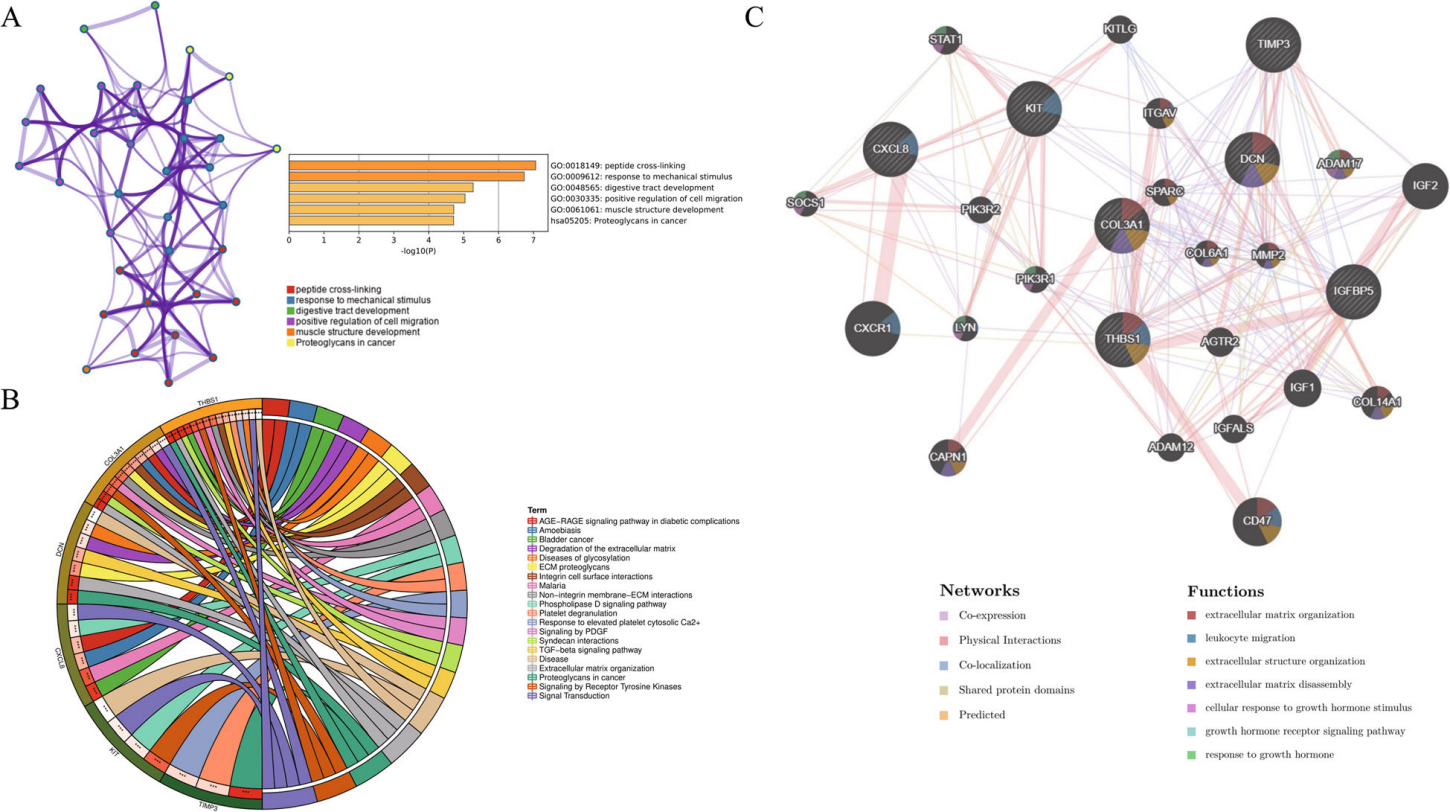
Biological process, pathway and interaction network analysis of the hub genes. **a** The top 5 enriched GO categories of biological process via Metascape. **b** The pathway analysis of the hub genes by KOBAS 3.0. The outermost circle is term on the right, the color corresponding to the gene on the left is the gene’s expression multiple, and the inner circle on the left represents the significant *P*-value of the corresponding pathway of the gene. **c** Hub genes and their co-expression genes were analyzed using GeneMANIA

**Fig. 5:**
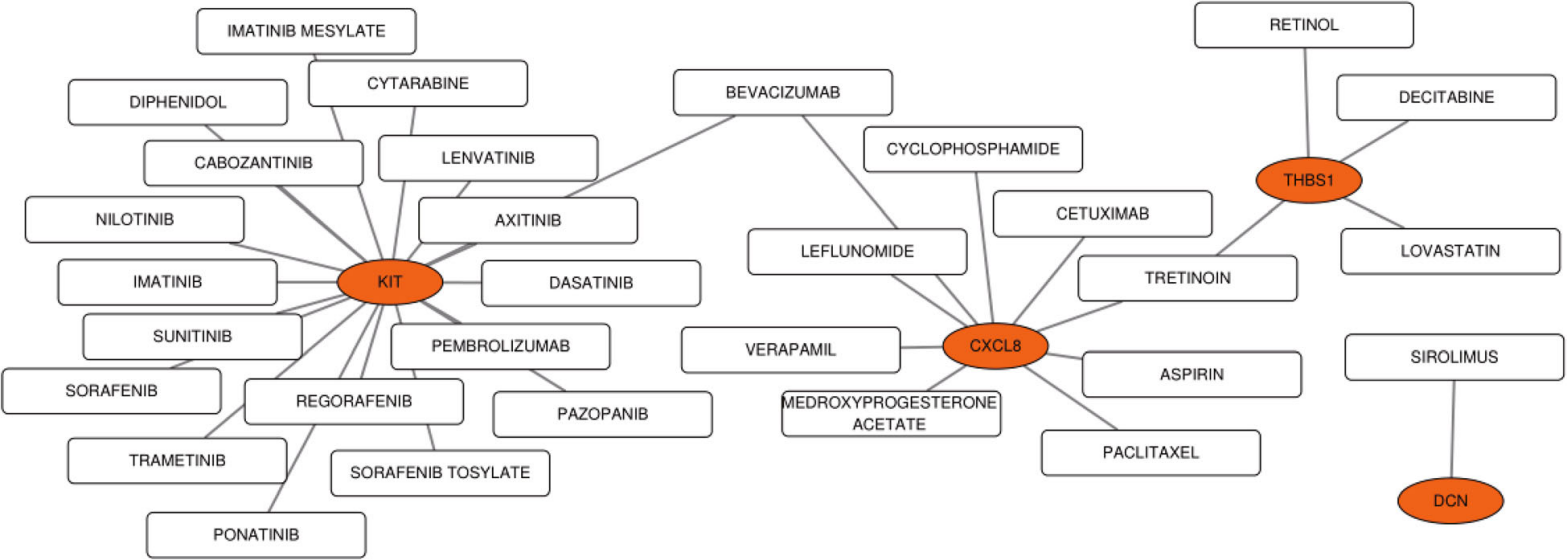
Based on the DGIdb predictions of the module genes, we obtained 32 drug-gene interaction pairs, including four upregulated genes (CXCL8, THBS1, KIT and DCN) and 30 drugs (FDA-listed + antitumor drugs). Yellow circle indicates the differentially expressed gene and blank square indicates the drug

The PPI network of DEGs with combined scores greater than 0.4 was generated by Cytoscape, which contained 68 nodes and 127 interaction pairs (Fig. 1b). A total of seven genes with degrees ≥10 was identified as hub genes. Detailed information on hub genes, including gene symbols, degrees, full names and gene function, was shown in Table 1. As expected, functional annotation obtained from Metascape suggested that hub genes were mainly enriched in peptide cross-linking, response to mechanical stimulus and regulation of vasculature development (Fig. 4a). The pathway analyses of the hub genes were conducted using KOBAS 3.0 and pathways with the top three *P*-value were proteoglycans in cancer (*P* = 1.67 × 10^− 6^), extracellular matrix organization (*P* = 5.2 × 10^− 6^) and syndecan interactions (*P* = 5.36 × 10^− 6^) (Fig. 4b). Similarly, these results emphasize the important role of proteoglycans and extracellular matrix organization in the metastasis of melanoma. Besides, a network of the hub genes and their co-expression genes was analyzed by GeneMANIA online platform. The seven genes showed the complex PPI network with the Co-expression of 44.42%, Physical interactions of 40.75%, Co-localization of 13.43%, Shared protein domains of 1.31% and Predicted of 0.09% (Fig. 4c). Finally, based on the DGIdb predictions of the hub genes, we obtained 32 drug-gene interaction pairs, including four upregulated genes (CXCL8, THBS1, KIT and DCN) and 30 drugs (FDA-listed + antitumor drugs), as shown in Fig. 5. These results may reveal the therapeutic targets related to metastatic melanoma.

**Table 1:** Details of seven hub genes

Gene symbols	Degrees	Full names	Gene function
CXCL8	17	C-X-C motif chemokine ligand 8	This chemokine is a potent angiogenic factor.
THBS1	13	thrombospondin 1	This protein has been shown to play roles in platelet aggregation, angiogenesis, and tumorigenesis.
COL3A1	12	collagen type III alpha 1 chain	Mutations in this gene are associated with Ehlers-Danlos syndrome types IV, and with aortic and arterial aneurysms.
TIMP3	11	TIMP metallopeptidase inhibitor 3	The proteins encoded by this gene family are inhibitors of the matrix metalloproteinases, a group of peptidases involved in degradation of the extracellular matrix (ECM).
KIT	11	KIT proto-oncogene receptor tyrosine kinase	Mutations in this gene are associated with gastrointestinal stromal tumors, mast cell disease, acute myelogenous lukemia and piebaldism.
DCN	10	decorin	Binding of this protein to multiple cell surface receptors mediates its role in tumor suppression, including a stimulatory effect on autophagy and inflammation and an inhibitory effect on angiogenesis and tumorigenesis.
IGFBP5	10	insulin like growth factor binding protein 5	IGF-binding proteins prolong the half-life of the IGFs and have been shown to either inhibit or stimulate the growth promoting effects of the IGFs on cell culture.

### Validation of hub genes expression in TCGA database

**Fig. 6:**
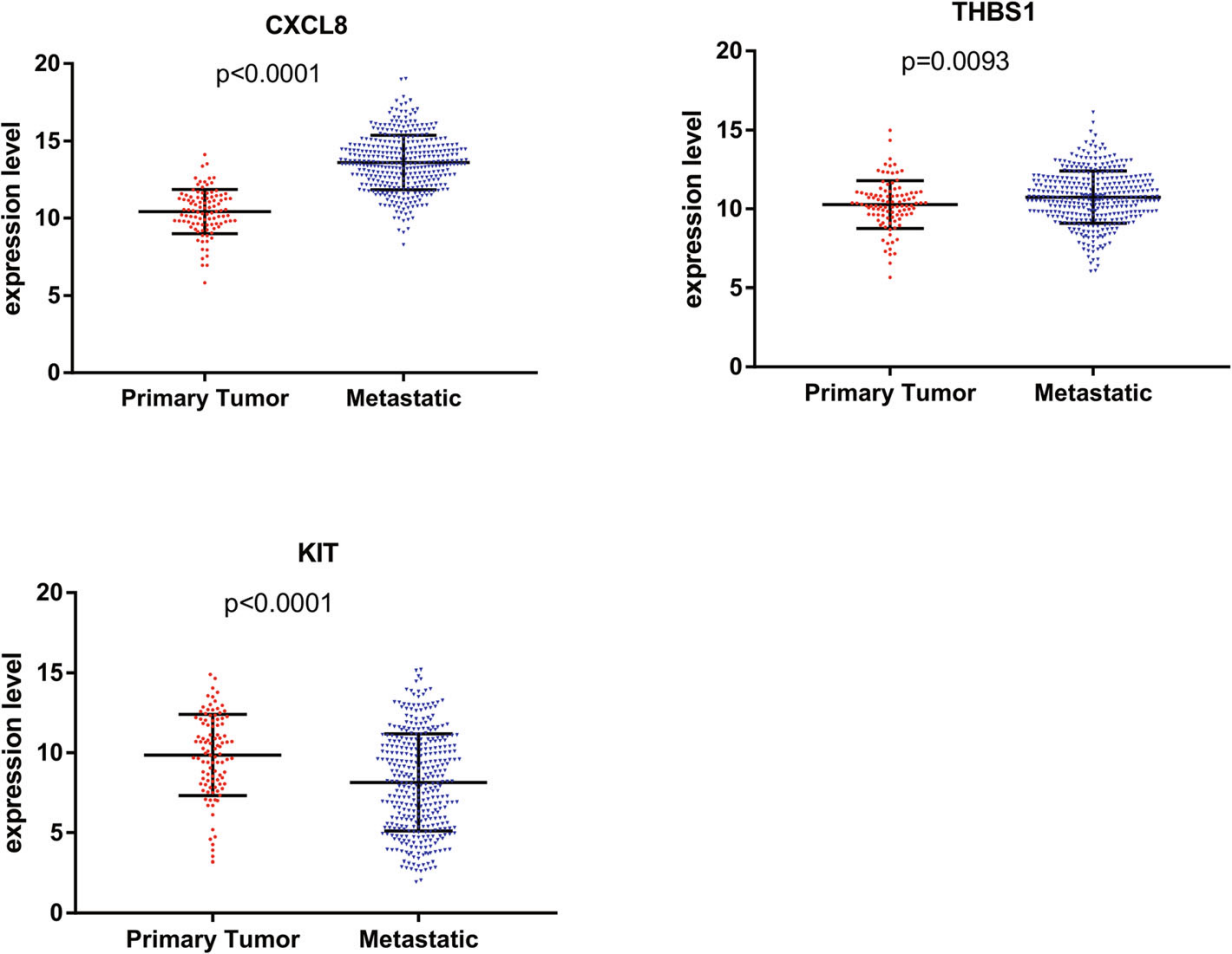
The mRNA expression level of hub genes in primary melanomas and metastatic melanomas was verified in TCGA database. The comparison between the two sets of data uses the mean T test. *P*-value < 0.05 was considered statistically significant

In order to prove the reliability and accuracy of the results of bioinformatics analysis, we checked the transcription level of the hub genes in the TCGA database, a platform for obtaining various cancer data. The results of independence testing analysis showed that genes of CXCL8 and THBS1 had a significant increase of gene expression in metastatic melanoma, but a significant downregulation of KIT expression (Fig. 6).

### Immunohistochemistry (IHC)

**Fig. 7:**
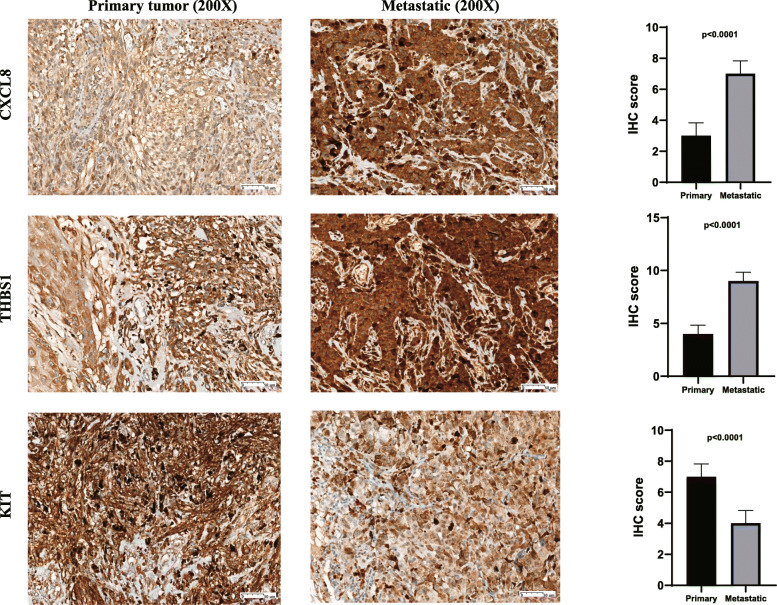
Validation of three hub genes expression in melanoma tissues from the clinical specimens. IHC staining indicated significantly elevated expression of CXCL8 and THBS1 protein in metastatic melanoma was significantly higher than that of primary melanoma, while the expression level of KIT was lower

In order to further explore the protein levels of the corresponding genes, we used the data obtained from clinical specimens to analyze the protein expression pattern of the hub genes in melanoma. IHC was used to verify the protein expression levels of the three hub genes. Consistent with mRNA expression, we found that the expression of CXCL8 and THBS1 protein in metastatic melanoma was significantly higher than that of primary melanoma, while the expression level of KIT was lower. The results and graphs of the IHC score are shown in Fig. 7.

## Discussion

In this study, a total of 41 up-regulated genes and 79 down-regulated genes were identified from three microarray data sets and thoroughly analyzed. Pathway analysis showed that these genes are mainly involved in extracellular matrix organization and proteoglycans in cancer. Several hub genes, CXCL8, THBS1, KIT, and DCN, were found in the PPI network, and interestingly, also found in the predicted drug-gene interactions. However, according to the independent test results of the TCGA database, the difference of CXCL8, THBS1 and KIT in mRNA expression changes was significant. In addition, it was verified in clinical samples that the expression level of the three genes was consistent with the mRNA expression pattern.

The extracellular matrix (ECM) performs many functions in addition to its structural role; as a major component of the cellular microenvironment it influences cell behaviors such as proliferation, adhesion and migration, and regulates cell differentiation and death [18]. Abnormal ECM dynamics can lead to deregulated cell proliferation and invasion, failure of cell death, and loss of cell differentiation, resulting in congenital defects and pathological processes including tissue fibrosis and cancer. Proteoglycans, as ECM constituents, is lost in aged fibroblasts, resulting in a more aligned ECM that promoted metastasis of melanoma cells [19].

CXCL8(interleukin-8) is considered to be a typical chemokine belonging to the CXC family, responsible for the recruitment and activation of neutrophils and granulocytes at the site of inflammation. Its role in the progression of melanoma mainly depends on its interaction with specific cell surface G protein coupled receptor (GPCR), C-X-C chemokine receptor type 1 (CXCR1) and C-X-C chemokine receptor type 2 (CXCR2) [20–22]. Varney et al. examined the expression of CXCL8, its receptors, CXCR1 and CXCR2, and vessel density in human melanoma by immunohistochemical analysis of tumors from different Clark levels, depths and thicknesses, and found that the expression of CXCL8 and CXCR2 was lower in Clark level I and II specimens than in level III through V specimens and metastases [23]. It indicates that the expression of CXCL8 and CXCR2 contributes to the aggressive growth and metastasis of human malignant melanoma. Three years later, a live mouse study demonstrated that CXCR2 plays a key role in melanoma lung metastasis through a gene knockout model [24]. In addition, Wu et al. evaluated the role of CXCL8 in the growth and progression of melanoma by regulating its expression in melanoma cell lines expressing different levels of CXCL8, and found that the expression of CXCL8 is a key in regulating multiple cell phenotypes associated with melanoma growth and metastasis [25]. It shows that CXCL8 is an important biomarker in the process of melanoma metastasis.

As a matricellular glycoprotein, THBS1 regulates cellular phenotype and extracellular structure during tissue genesis and remodeling, and has been shown to regulate tumor progression and metastasis [26, 27]. There is increasing evidence that the acquisition of invasive and metastatic features of melanoma cells involves the reactivation of a developmental EMT-like program [28–30]. More importantly, the results of the biological pathway enrichment of DEGs in the study also confirmed this conclusion. As the main physiological activator of transforming growth factor-β (TGF-β), THBS1 may activate the latent TGF-β1 in the progress of melanoma to promote EMT of melanoma [31–33]. Another study also validated that increased expression of THBS1 is associated with an invasive and metastatic phenotype of melanoma, as part of a Slug-independent motility program that includes the melanoma-related VEGF/VEGFR-1 and FGF-2 pathways [34]. In addition, THBS1 has been shown to promote cell invasion of breast cancer, thyroid cancer, colon cancer and prostate cancer. Therefore, we can draw a clear conclusion that THBS1 promotes the invasion and metastasis of melanoma, which is expected to become a target for future treatment.

KIT, a tyrosine kinase receptor encoding stem cell factor, plays an important role in the development, migration and proliferation of melanocytes [35, 36]. Although KIT is expressed in some melanomas, as the disease progresses from the superficial stage to infiltration and then to the metastasis stage, the loss of KIT expression indicates that KIT has tumor suppressive function [37–39]. A recent study also found that in patients without lymph node metastasis at the initial diagnosis, the expression of KIT was significantly higher than that of patients with lymph node metastasis, indicating that melanoma with missing KIT expression is more likely to progress and metastasize [40]. In addition, KIT is the target of several small molecule inhibitors such as imatinib and nilotinib. These drugs have been used clinically and can significantly extend the lifespan of patients with metastatic melanoma carrying KIT mutations [41, 42]. Therefore, we believe that it mediates the metastasis of melanoma and can be used as a target for the treatment of metastatic melanoma [43].

In this study, we highlighted the potential role of CXCL8, THBS1 and KIT in melanoma metastasis. However, we acknowledged that the study has some certain limitations. Although we have verified the differences in mRNA and protein expression levels of these genes in TCGA databases and clinical specimens, in our future studies, the biological function of these genes in melanoma needs further study.

## Conclusions

In summary, the purpose of this study was to identify DEGs that may be associated with melanoma metastasis. A total of three hub genes have been identified, which can be used as a biomarker for metastatic melanoma or as a drug therapy target.

## Data Availability

In this study, mRNA microarray datasets were downloaded from the GEO (http://www.ncbi.nlm.nih.gov/geo) and TCGA (https://tcga-data.nci.nih.gov/tcga/) database.

## Abbreviations

GEO: Gene expression omnibus
DEG: Differentially expressed gene
PPI: Protein-protein interaction
DGIdb: Drug-Gene interaction database
BP: Biological processes
CC: Cell component
MF: Molecular function
EMT: Epithelial-to-mesenchymal transition
CXCL8: C-X-C motif chemokine ligand 8
THBS1: Thrombospondin 1
COL3A1: Collagen type III alpha 1 chain
TIMP3: TIMP metallopeptidase inhibitor 3
KIT: KIT Proto-oncogene receptor tyrosine kinase
DCN: Decorin
IGFBP5: Insulin like growth factor binding protein 5
ECM: Extracellular matrix
GPCR: G protein coupled receptor
CXCR1: C-X-C chemokine receptor type 1
CXCR2: C-X-C chemokine receptor type 2
TGF-β: Transforming growth factor-β
